# Synthesis, Biological, Spectral, and Thermal Investigations of Cobalt(II) and Nickel(II) Complexes of N-Isonicotinamido -2′,4′-Dichlorobenzalaldimine

**DOI:** 10.1155/BCA/2006/29234

**Published:** 2006-06-06

**Authors:** Ram K. Agarwal, Deepak Sharma, Lakshman Singh, Himanshu Agarwal

**Affiliations:** ^1^Department of Chemistry, School of Pure and Applied Sciences, The University of the South Pacific, PO Box 1168, Suva, Fiji; ^2^Department of Chemistry, Lajpat Rai Postgraduate College, Sahibabad 201005, India

## Abstract

A new series of 12 complexes of cobalt(II) and nickel(II) with N-isonicotinamido-2′,4′-dichlorobenzalaldimine (INH-DCB) with the general composition MX_2_ · n(INH-DCB) [M = Co(II) or Ni(II), X = Cl^−^ ,Br^−^ , NO_3_^−^ , NCS^−^ , or CH_3_COO^−^ , n = 2; X = ClO_4_^−^, n = 3] have been synthesized. The nature of bonding and the stereochemistry of the complexes have been deduced from elemental analyses, infrared, electronic spectra, magnetic susceptibility, and conductivity measurements. An octahedral geometry has been suggested for all the complexes. The metal complexes were screened for their antifungal and antibacterial activities on different species of pathogenic fungi and bacteria and their biopotency has been discussed.

## INTRODUCTION

**Figure 1:**
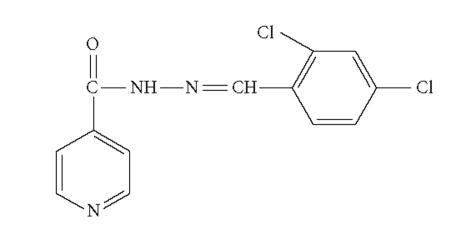
N-isonicotinamido-2′,4′-dichlorobenzaladimine (INH-DCB).

Interest in the study of hydrazones has been growing because of their antimicrobial, antituberculosis, and antitumour activity [1–8]. Hydrazones derived from condensation of isonicotinic acid hydrazide with pyridine aldehydes have been found to show better antitubercular activity than INH [9]. The remarkable biological activity of acid hydrazides R−CO−NH−NH_2_, their corresponding aroylhydrazones R−CO−NH−N=CH−R′, and the dependence of their mode of chelation with transition metal ions present in the living system have been of significant importance in the past [10–13]. In view of the versatile importance of hydrazones, we herein describe the synthesis and identification of the Co(II) and Ni(II) complexes of N-isonicotinamido-2′,4′-dichlorobenzalaldimine(INH-DCB) (Figure 1).

## EXPERIMENTAL

MX_2_ · nH_2_O (M = Co^2+^ or Ni^2+^ ; X = Cl^−^ , Br^−^ , NO_3_^−^ or CH_3_COO^−^ ) were obtained from SD Fine Chemicals Ltd (Mumbai, India) and were used as such: M(NCS)_2_ (M = Co^2+^ or Ni^2+^ ). They were prepared by mixing metal chloride (in ethanol) and ethanolic solution of potassium thiocyanate in 1 : 2 molar ratio. Precipitated KCl was filtered off and the filtrate having respective metal thiocyanate was used immediately for complex formation [14]. M(ClO_4_)_2_ (M = Co^2+^ or Ni^2+^ ) were prepared by the addition of an ethanolic solution of sodium perchlorate to respective metal chloride solution. White precipitate of NaCl was filtered off and the filtrate containg M(ClO_4_)_2_ was used as such for complex formation. The ligand INH-DCB was synthesized in the laboratory by the following method. Isonicotinic acid hydrazide (INH) (0.01 mol) was dissolved in 10 mL of 95% ethanol. To this solution, 2,4-dichlorobenzaldehyde (0.01 mol) was added in 95% ethanol (10 mL). The mixture was refluxed on a water bath for 1-2 hours. The partial removal of solvent on a water bath followed by cooling produced crystalline product, which was collected by filtration, washed with cold ethanol, and dried under vacuum (yield 80%). The purity of the ligand was checked by TLC, IR spectra, and melting point.

### Synthesis of the complexes

A general method has been used for the preparation of all the complexes. A hot ethanolic solution of the corresponding cobalt(II) or nickel(II) salt was mixed with a hot ethanolic solution of the ligand (in 1 : 2 or 1 : 3 molar ratio). The reaction mixture was refluxed on water bath for about 2-3 hours. On cooling at room temperature, the coloured complexes precipitated out in each case. They were filtered, washed with ethanol and recrystallized, and dried over P_2_O_5_ under vacuum.

### Physical measurements and analytical estimations

The cobalt(II) and nickel(II) ions in their metal complexes were estimated complexometrically with EDTA using murexide and erichrome black-T as an indicator after decomposing the complexes with concentrate H_2_SO_4_ and H_2_O_2_ [15]. The halogens and thiocyanate were estimated by Volhard's method [16]. The perchlorate was estimated by the method suggested by Kurz et al [17]. The nitrogen content was determined by Kjeldahl method. The molecular weight of the complexes was determined in laboratory cryoscopically in freezing nitrobenzene using a Beckmann thermometer of ±0.01°C accuracy. The conductivity measurements were carried out, at room temperature in nitrobenzene, using a conductivity bridge and dip-type cell operated at 220 volts AC mains. The magnetic measurements on powder form of the complexes were carried out at room temperature on Evans's balance using anhydrous copper(II) sulfate as calibrant. The infrared spectra of the complexes were recorded on a Perkin Elmer infrared spectrophotometer model Spectrum 1000 in CsI in the range of 200–4000 cm^−1^. Diffused reflectance spectra of the solid compounds were recorded on a Beckmann DK-2A spectrophotometer at CDRI, Lucknow, India. Thermogravimetric studies of the complexes were carried out in static air with open sample holder and a small boat, the heating rate was 6 °C/min.

Antibacterial activity was done by the paper-disc plate method. The nutrient agar medium (peptone, beef extract, NaCl, and agar-agar) and 5 mm diameter paper discs (Whatman number 1) were used. The compounds were dissolved in DMSO in 500 and 1000 ppm concentrations. The filter paper discs were soaked in different solutions of the compounds, dried, and then placed in the petri dishes previously seeded with the test organisms (*Escherichia coli* and *Klebseila aerogenous*). The plates were incubated for 24–30 hours at 28 ± 2°C and the inhibition zone around each disc was measured. The antifungal activity was evaluated against *Fusarium oxysporum* and *Macrophomina phaseolina* using standard food poisoning technique and a procedure recommended for testing new chemicals [18]. The linear growth of the fungus was recorded by measuring the diameter of the fungus colony after 96 hours and the percentage inhibition was calculated as 100 (C-T)/C, where C and T are the diameters of the fungus colony in the control and test plates, respectively.

### Results and discussion

The reaction of cobalt(II) and nickel(II) salts with INH-DCB results in the formation of MX_2_ · (INH-DCB)_*n*_ [M = Co(II) or Ni(II); X = Cl^−^ , Br^−^ , NO_3_^−^ , NCS^−^ , or CH_3_COO^−^ , n = 2; X = ClO_4_^−^, n = 3] (Table 1). All the complexes are quite stable and could be stored for months without any appreciable change. The complexes do not have sharp melting points but decompose above 250°C. These complexes are generally soluble in common organic solvents. The conductance measurement indicates that the chloro, bromo, nitrato, thiocyanato, and acetato complexes of cobalt(II) and nickel(II) are essentially nonelectrolytes in nitrobenzene, while the perchlorato complexes dissociate in nitrobenzene and behave as 1 : 2 electrolytes [19]. The molecular weights determined cryscopically are in broad agreement with the conductance data (Table 1).

### Magnetic susceptibility

The observed magnetic moments of cobalt(II) complexes of INH-DCB are given in Table 1. The theory of magnetic susceptibility of cobalt(II) ion was given originally by Schlapp and Penney [20] and the best summary of results on the magnetic behaviour of cobalt compound is that of Figgis and Nyholm [21]. The observed values of magnetic moment for cobalt(II) complexes are generally diagnostic of the coordination geometry about the metal ion. The low-spin square-planar cobalt(II) complexes may be 2.9 BM, arising from one unpaired electron plus an apparently large orbital contribution [21]. Both tetrahedral and high-spin octahedral cobalt(II) complexes possess three unpaired electrons but may be distinguished by the magnitude of the deviation of μ_eff_ from the spin-only value. The magnetic moment of tetrahedral cobalt(II) complexes with an orbitally nongenerate ground term is increased above the spin-only value via contribution from higher orbitally degenerate terms and occurs in the range 4.2–4.7 BM [22]. Octahedral cobalt(II) complexes however maintain a large contribution due to ^4^ T_g_ ground term and exhibit μ_eff_ in the range 4.8–5.6 BM [23]. The magnetic measurements on the complexes reported herein 4.7–5.1 BM show that all are paramagnetic and have three unpaired electrons indicating a high-spin octahedral configuration.

Magnetic behavior of octahedral nickel(II) complexes is relatively simple. Nickel(II) has the electronic configuration 3*d* ^8^ and should exhibit a magnetic moment higher than expected for two unpaired electrons in octahedral (2.8–3.2 BM) and tetrahedral (3.4–4.2 BM) complexes whereas its square-planar complexes would be diamagnetic. This increase in the magnetic moment value from that of the spin-only value has been discussed by Nyholm [24] who considered it to be due to some “mixing in” of upper state via spin-orbit coupling. The paramagnetism observed for the present series of complexes ranges from 2.6–3.2 BM (Table 1) which is consistent with the octahedral stereochemistry of the complexes.

### Infrared spectra

INH-DCB is expected to act as tridentate one, the possible coordination sites being pyridinic-nitrogen, azomethine-nitrogen, and amide group. A study and comparison of the IR spectra of INH-DCB and its cobalt(II) and nickel(II) complexes imply that the ligand INH-DCB is bidentate in nature with carbonyl-oxygen and azomethine-nitrogen as two coordination sites. The IR-data are presented in Table 2.

Generally, all amides show two absorption bands, (i) the carbonyl absorption band near 1640 cm^−1^ known as amide-I band and (ii) strong band in the 1500–1600 cm^−1^ region, known as amide-II band. The origin of these bands in hydrazones, that is, the carbonyl absorption responsible for the amide-I band, is likely to be lowered [25] infrequently by the NH group as in normal amides. The amide-I band in INH-derivative, however, appears at 1700 and 1655 cm^−1^ [26, 27]. In the IR spectra of the complexes a considerable negative shift in *ν*(C=O) is observed indicating a decrease in the stretching force constant of C=O as a consequence of coordination through the carbonyl-oxygen atom of the free base. The amide-II band appears at the normal position in the NH-deformation rather than the C−N link. In all the hydrazones, the absorptions such as 1540, 1520 cm^−1^ have been assigned to amide-II absorption. The NH stretching absorption in free ligand occurs at ∼ 3300 and 3220 cm^−1^ [28] which remains unaffected after complexation. This precludes the possibility of coordination through imine nitrogen atom.

Another important band occurs at ∼ 1585 cm^−1^ attributed to *ν*(C=N) (azomethine) mode [29–31]. In spectra of all the complexes this band is shifted to lower wave number and appears in 1525–1555 cm^−1^ region, respectively, indicating the involvement of N-atom of the azomethine group in coordination [32–34].

The strong bands observed at 1520–1575 cm^−1^ and 1000–1080 cm^−1^ are tentatively assigned [29, 30, 35] to asymmetric and symmetric *ν*(C=C) + *ν*(C=N) of pyridine ring and pyridine ring breathings and deformations remain practically unchanged in frequency and band intensities revealing noninvolvement of pyridinic-nitrogen and metal bond. The overall IR spectral evidence suggests that the INH-DCB acts as bidentate ligand and coordinate through amide-oxygen and azomethine-nitrogen atoms forming a five-membered chelate ring. In the far IR spectral region, the bands in the ligand are practically unchanged in these complexes. However some new bands with medium to weak intensities appear in the regions 395–505 cm^−1^ in the complexes under study, which are tentatively assigned to *ν*(M−O)/*ν*(M−N) modes [25].

### Anions

In both perchlorato complexes, the presence of the *ν*_3_ at ∼ 1100 cm^−1^ and *ν*_4_ at ∼ 625 cm^−1^ bands indicates that the T_d_ symmetry of ClO_4_^−^ is maintained in all the complexes. This, therefore, suggests the presence of ClO_4_^−^ outside the coordination sphere in the complexes [31, 36, 37]. The CN stretching frequency (*ν*_1_) is generally lower for M-SCN complexes than for M-SCN complexes [38]. Bailey et al [39] suggested the region near or above 2100 cm^−1^ for S-bonding, below this for N-bonding. The CS stretching frequency (*ν*_2_) was assigned in the following regions: 780–860 cm^−1^ for M-SCN and 690–720 cm^−1^ for M-SCN group [40]. The NCS frequency (*ν*_3_) is also different for the two isomers 450–490 cm^−1^ for the M-SCN and 400–440 cm^−1^ for M-SCN group [40]. Bridging thiocyanate usually gives higher CN stretching frequencies than terminal NCS group [41–43]. In present thiocyanato complexes, three fundamental absorptions C−N stretch (*ν*_1_), C−S stretch (*ν*_3_), and N−C−S bending (*ν*_2_) are identified at ∼ 2050, 840, and 475, respectively. These frequencies are associated with the terminal N-bonded isothiocyanate ions [41–43]. The occurrence of two strong absorption bands in both the nitrato complexes at ∼ 1500 and 1300 cm^−1^ are attributed to *ν*_4_ and *ν*_1_ modes of vibrations of the covalently bonded nitrate groups, respectively. This suggests that nitrate groups are present inside the coordination sphere [44, 45]. If the (*ν*_4_ − *ν*_1_) difference is taken as an approximate measure of the covalency of nitrate group [46, 47], a value of ∼ 200 cm^−1^ for the complexes studied suggests strong covalency for the metal-nitrate bonding. Devi et al [48] have shown that the number and relative energies of nitrate combination frequencies (*ν*_1_ + *ν*_4_) in the 1700–1800 cm^−1^ region of the infrared spectrum may be used as an aid to distinguish the various coordination modes of the nitrato group. According to Agarwal et al [49], bidentate coordination involves a greater distortion from D_3h_ symmetry than unidentate coordination, therefore, bidentate complexes should show a larger separation of (*ν*_1_ + *ν*_4_). By an investigation of the spectra of a number of compounds of known crystal structure, Devi et al [48] showed this to be true, the separation of monodentate nitrate groups appeared to be 5–26 cm^−1^ and that for bidentate groups 25–66 cm^−1^. The authors have tried to apply this method to present complexes. In both cases, in all the nitrato complexes, a separation of 15–25 cm^−1^ in the combination bands (*ν*_1_ + *ν*_4_) in the 1700–1800 cm^−1^ region conclude the monodentate nitrate coordination.

The *ν*_asym_ (COO^−^ ) of free acetate ions are at ∼ 1560 cm^−1^ and 1416 cm^−1^, respectively. In the unidentate complex (structure a) *ν*(C=O) is higher than *ν*_asym_ (COO^−^ ) and *ν*(C−O) is lower than *ν*_asym_ (COO^−^ ). As a result, the separation between the two *ν*(CO) is much larger in unidentate complexes than that of free ion. The opposite trend is observed in the bidentate complex, the separation between the *ν*(CO) is smaller than that of free ion in this case. In the bridging complexes (structure c), however, two *ν*(CO) are close to the free ion values. The present complexes show infrared absorption frequency bands corresponding to *ν*_asym_ (COO^−^ ) and *ν*_sym_ (COO^−^ ) at ∼ 1610 and 1370 cm^−1^, respectively. These observations indicate that both the acetate groups in present complexes are unidentate [50, 51].

### Electronic spectra

#### Cobalt(II) complexes

The electronic spectra of all the present cobalt(II) complexes recorded herein are very similar to each other and consist of two bands one in the 15, 400–15, 500 cm^−1^ and the other in the 20, 500–20, 830 cm^−1^ regions, which clearly indicate the octahedral stereochemistry of the complexes. In Table 3, the band maxima and their assignments and the calculated ligand field parameters are listed. When all the bands, *ν*_1_, *ν*_2_, and *ν*_3_ are observed to be free from shoulders, the ligand field parameters D_q_ and B are, in principle, calculated using first-order perturbation theory [52, 53] and the transition energies are given by the following equations [54]:

(1) $$\nu_{1}=5{\text{D}}_{\text{q}}-7.58+\frac{1}{2}{\left(225{\text{B}}^{2}+100{\text{D}}_{\text{q}}{\;}^{\text{2}}{\text{+180D}}_{\text{q}}\text{B}\right)}^{1/2},$$

$$\nu_{2}=15{\text{D}}_{\text{q}}-7.58+\frac{1}{2}(225{\text{B}}^{2}+100{\text{D}}_{\text{q}}{\;}^{\text{2}}{\text{+180D}}_{\text{q}}\text{B}),$$

$$\nu_{3}={(225\text{B}+100{\text{D}}_{\text{q}}+180{\text{D}}_{\text{q}}\text{B)}}^{\text{1/2}}$$.

The methods of calculation of ligand field parameters from the ligand field spectra of octahedral Co(II) complexes have been discussed by Reedijk et al [55]. The energy of *ν*_1_ corresponds to 10D_q_ for weak field and the value of D_q_ is obtained from it. With these assignments, B and D_q_ have also been observed (Table 3).

#### Nickel(II) complexes

The electronic spectra of all the complexes recorded herein are very similar to each other and consist of three bands one at ∼ 10 000 cm^−1^ due to ^3^ A_2g_ → ^3^ T_2g_ (*ν*_1_), ∼ 16 000 cm^−1^ due to ^3^ A_2g_ → ^3^ T_1g_ (*ν*_2_), and ∼ 25 000 cm^−1^ for ^3^ A_2g_ → ^3^ T_2g_ (*ν*_3_) which clearly indicate the octahedral stereochemistry of the complexes. In Table 4, the band maxima and their assignments and the calculated ligand field parameters are listed [52–54].

### Thermal studies

The thermal results of Co(II) and Ni(II) complexes of INH-DCB are briefed in Tables 5 and Table 6, respectively. Due to the explosive nature of perchlorato complexes, we have investigated only the thermal properties of chloro, bromo, and nitrato complexes.All the complexes are thermally stable up to 165°C. After that deligation process started and in temperature range 165–270°C, one mol of INH-DCB is lost, which is confirmed by mass loss of 37.20%–41.96% at this stage. Another mol of INH-DCB is lost in the 280–390°C temperature range. Finally at ∼ 615°C, metal-oxide (Co_3_O_4_ or NiO) formation takes place [49].

### Biological properties

The antimicrobial screening data are presented in Table 7. The table shows that the metal complexes exhibit antimicrobial properties and it is important to note that these complexes exhibit enhanced activity in contrast to the free ligand. The increased lipophilic character of these complexes seems to be responsible for their enhanced potent antibacterial activity. It may be suggested that these complexes deactivate various cellular enzymes, which play a vital role in various metabolic pathways of these microorganisms. It has also been proposed that the ultimate action of the toxicant is the denaturation of one or more proteins of the cell, which as a result, impairs normal cellular processes. The antifungal activity of the cobalt(II) and nickel(II) complexes was evaluated against *F oxysporum* and *M phaseolina* by the agar plate techniques by mixing solutions of the metal complexes in different concentrations in DMF which were then mixed with the medium. The linear growth of the fungus was recorded by measuring the diameter of colony after 96 hours and the percentage inhibition was calculated as 100 (C-T)/C, where C and T are the diameters of the fungus colony in the control and test plates, respectively (Table 8).

## CONCLUSION

**Figure 2:**
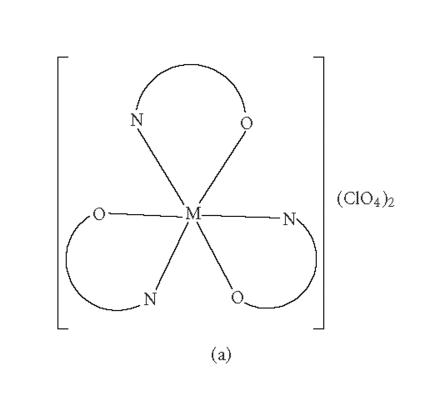

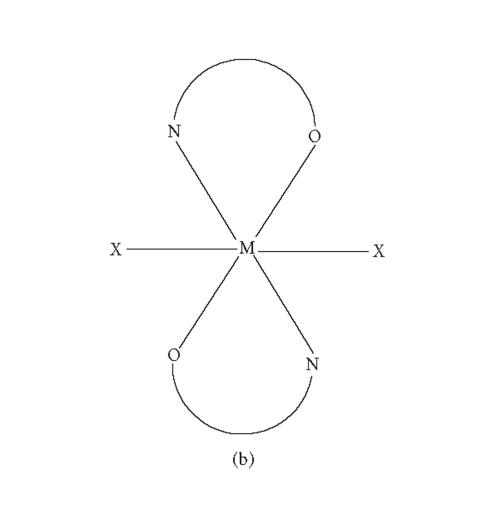
(a) Suggested structure of [M(INH-DCB)_3_] (ClO_4_)_2_ (M = Co^2+^ or Ni^2+^ ); (b) suggested structure of [M(INH-DCB)_2_ X_2_ ] (M = Co^2+^ or Ni^2+^ ; X = Cl^−^ , Br^−^ , I^−^ , NCS^−^ , or NO_3_^−^ ).

The present study revealed octahedral geometry around Co(II) and Ni(II) complexes, in which the ligand INH-DCB acts as a neutral bidentate coordinating through nitrogen and oxygen atoms and thus forming stable five-membered chelates. Tentative structures of the present chelates can be shown in Figures 2(a) and 2(b). The results of antimicrobial activity show that the metal complexes exhibit antimicrobial properties and it is important to note that they show enhanced inhibitory activity compared to the parent ligand. It has also been proposed that concentration plays a vital role in increasing the degree of inhibition; as the concentration increases, the activity increases.

## Figures and Tables

**Table 1:** Analytical conductivity, molecular weight, and magnetic data of Co^2+^ and Ni^2+^ complexes of INH-DCB.

Complex	Yield (%)	Analysis: found (calcd) (%)					Mol wt	Ω_M_ (Ohm cm^2^ mol^−1^)	μ_eff_ (BM)
							found		
							(calcd)		

		Metal	C	H	N	Anion

CoCl2 · 2(INH-DCB)	72	8.16 (8.21)	43.23 (43.45)	2.46 (2.50)	11.58 (11.69)	9.79 (9.88)	714 (718)	1.9	5.1
CoBr_2_ · 2(INH-DCB)	68	7.27 (7.31)	38.49 (38.66)	2.19 (2.23)	10.30 (10.40)	19.65 (19.82)	804 (807)	2.4	4.9
Co(NO_3_)_2_ · 2(INH-DCB)	75	7.60 (7.65)	40.68 (40.96)	2.28 (2.33)	14.40 (14.52)	—	765 (771)	1.8	4.7
Co(NCS)_2_ · 2(INH-DCB)	70	7.68 (7.73)	43.79 (44.03)	2.30 (2.35)	14.55 (14.67)	15.08 (15.20)	758 (763)	2.3	5.0
Co(CH_3_COO)_2_ · 2(INH-DCB)	70	7.65 (7.71)	46.77 (47.05)	3.09 (3.13)	10.87 (10.98)	—	760 (765)	1.9	4.8
Co(ClO_4_)_2_ · 3(INH-DCB)	65	5.09 (5.17)	40.79 (41.05)	2.32 (2.36)	10.95 (11.05)	17.34 (17.45)	380 (1140)	51.9	4.9
NiCl_2_ · 2(INH-DCB)	70	8.17 (8.21)	43.20 (43.45)	2.46 (2.50)	11.09 (11.69)	9.78 (9.88)	713 (718)	2.1	3.1
NiBr_2_ · 2(INH-DCB)	68	7.28 (7.31)	38.49 (38.66)	2.19 (2.23)	10.00 (10.40)	19.63 (19.82)	800 (807)	2.2	2.9
Ni(NO_3_)_2_ · 2(INH-DCB)	72	7.61 (7.65)	40.78 (40.96)	2.29 (2.33)	14.13 (14.52)	—	765 (771)	1.8	3.2
Ni(NCS)_2_ · 2(INH-DCB)	70	7.68 (7.73)	43.79 (44.03)	2.30 (2.35)	14.16 (14.67)	14.98 (15.20)	758 (763)	2.3	2.6
Ni(CH_3_COO)_2_ · 2(INH-DCB)	75	7.66 (7.71)	46.81 (47.05)	3.10 (3.13)	10.79 (10.98)	—	760 (765)	1.9	2.8
Ni(ClO_4_)_2_ · 3(INH-DCB)	68	5.09 (5.17)	40.89 (41.05)	2.32 (2.36)	10.92 (11.05)	17.38 (17.45)	381 (1140)	50.9	3.2

**Table 2:** Infrared absorption frequencies (cm^−1^) of Co^2+^ and Ni^2+^ complexes INH-DCB.

Complex	*ν*(NH)	Amide-I	*ν*(C=N) azomethinic	*ν*(M−N)/*ν* (M−O)

INH-DCB	3300 m	1700 vs	1585 s	—
	3220 w	1655 vs		
CoCl_2_ · 2(INH-DCB)	3305 m	1670 vs	1525 m	490 m, 398 w
	3220 w	1605 vs		
CoBr_2_ · 2(INH-DCB)	3302 m	1670 s	1530 vs	492 m, 402 w
	3220 w	1610 vs, 1580 m		
Co(NO_3_)_2_ · 2(INH-DCB)	3300 m	1680 s	1555 s	502 m, 398 w
	3220 w	1620 s		
Co(NCS)_2_ · 2(INH-DCB)	3302 m	1670 vs	1525 m	505 m, 400 w
	3220 w	1620 vs, br		
Co(CH_3_COO)_2_ · 2(INH-DCB)	3300 m	1670 vs	1530 s	499 m, 402 w
	3220 w	1600 vs, br		
Co(ClO_4_)_2_ · 3(INH-DCB)	3300 m	1660 s	1532 s	498 m, 398 w
	3225 w	1605 s		
NiCl_2_ · 2(INH-DCB)	3305 m	1660 vs	1530 s	490 m, 398 w
	3220 w	1605 vs		
NiBr_2_ · 2(INH-DCB)	3302 m	1670 s	1555 m	495 m, 395 w
	3220 w	1600 vs, br		
Ni(NO_3_)_2_ · 2(INH-DCB)	3300 m	1680 s	1525 m	502 m, 398 w
	3222 w	1620 s		
Ni(NCS)_2_ · 2(INH-DCB)	3300 m	1670 s	1530 sh	505 m, 400 w
	3222 w	1605 vs		
Ni(CH_3_COO)_2_ · 2(INH-DCB)	3305 m	1660 s	1525 m	500 m, 402 w
	3220 w	1605 s		
Ni(ClO_4_)_2_ · 3(INH-DCB)	3300 m	1662 s	1530 m	505 m, 410 w
	3220 w	1660 vs		

**Table 3:** Electronic spectral bands (cm^−1^) and ligand-field parameters of Co^2+^ complexes of INH-DCB.

Complex	*ν*_2_	*ν*_3_	Dq	B	β	Dq/B	*ν*_1_
	^4^ T_1g_(F) → ^4^ A_2g_	^4^ T_1g_(F) → ^4^ T_1g_(P)	(cm^−1^)	(cm^−1^)			(cm^−1^)

CoCl_2_ · 2(INH-DCB)	15500	20830	861	956	0.853	0.90	7955
CoBr_2_ · 2(INH-DCB)	15450	20670	858	953	0.850	0.90	7806
Co(NCS)_2_ · 2(INH-DCB)	15400	20500	855	950	0.848	0.90	7836
Co(NO_3_)_2_ · 2(INH-DCB)	15500	20830	861	956	0.853	0.90	7955
Co(CH_3_COO)_2_ · 2(INH-DCB)	15400	20500	855	950	0.848	0.90	7830
Co(ClO_4_)_2_ · 3(INH-DCB)	15500	20830	861	956	0.853	0.90	7955

**Table 4:** Electronic spectral bands (cm^−1^) and ligand-field parameters of Ni^2+^ complexes of INH-DCB.

Complex	*ν*_1_	*ν*_2_	*ν*_3_	Dq (cm^−1^)	B (cm^−1^)	β

NiCl_2_ · 2(INH-DCB)	9090	15150	25000	909	988	0.91
NiBr_2_ · 2(INH-DCB)	9600	16200	24400	960	1043	0.96
Ni(NO_3_)_2_ · 2(INH-DCB)	9900	16660	24390	990	1076	0.99
Ni(NCS)_2_ · 2(INH-DCB)	9800	16700	24500	980	1065	0.98
Ni(CH_3_COO)_2_ · 2(INH-DCB)	9600	15385	25640	960	1043	0.96
Ni(ClO_4_)_2_ · 3(INH-DCB)	9900	16660	24390	990	1076	0.99

**Table 5:** Thermoanalytical results obtained for Co^2+^ of INH-DCB.

Complex	Decomp temp (°C)		Decomp product	Weight loss (%)	
	Initial	Final		Theor	Exp

Co(INH-DCB)_2_ · Cl_2_	180	250	Co(INH-DCB)Cl_2_	40.94	41.96
	300	360	CoCl_2_	81.89	82.91
	500	600	Co_3_O_4_	88.81	89.62
Co(INH-DCB)_2_ · Br_2_	165	235	Co(INH-DCB)Br_2_	36.43	37.20
	280	370	CoBr_2_	72.86	74.01
	505	610	Co_3_O_4_	90.04	91.26
Co(INH-DCB)_2_ (NO_3_)_2_	200	260	Co(INH-DCB)(NO_3_)_2_	38.13	39.86
	320	390	Co(NO_3_)_2_	76.26	77.36
	500	610	Co_3_O_4_	89.58	90.34

**Table 6:** Thermoanalytical results obtained for Ni^2+^ of INH-DCB.

Complex	Decomp temp (°C)		Decomp product	Weight loss (%)	
	Initial	Final		Theor	Exp

Ni(INH-DCB)_2_ Cl_2_	190	245	Ni(INH-DCB)Cl_2_	40.94	41.62
	290	365	NiCl_2_	81.80	82.56
	505	610	NiO	89.55	90.32
Ni(INH-DCB)_2_ Br_2_	175	235	Ni(INH-DCB)Br_2_	36.43	37.38
	285	375	NiBr_2_	72.86	73.42
	510	615	NiO	90.70	91.35
Ni(INH-DCB)_2_ (NO_3_)_2_	210	270	Ni(INH-DCB)(NO_3_)_2_	38.13	39.26
	300	390	Ni(NO_3_)_2_	76.26	77.16
	500	605	NiO	90.27	91.32

**Table 7:** Antibacterial screening data of INH-DCB and its Co(II) and Ni(II) complexes.

Compound	Diameter of inhibition zone			
	(mm) (conc in ppm)			
	*E coli*		*K aerogenous*	
	500	1000	500	1000

INH-DCB	6	8	5	8
CoCl_2_ · 2(INH-DCB)	9	11	8	10
CoBr_2_.2(INH – DCB)	9	10	8	11
Co(NO_3_)_2_ · 2(INH-DCB)	10	12	9	11
Co(NCS)_2_ · 2(INH-DCB)	11	13	10	12
Co(CH_3_COO)_2_ · 2(INH-DCB)	10	12	10	12
Co(ClO_4_)_2_ · 3(INH-DCB)	10	12	9	11
NiCl_2_ · 2(INH-DCB)	8	10	9	11
NiBr_2_ · 2(INH-DCB)	8	10	7	8
Ni(NO_3_)_2_ · 2(INH-DCB)	9	11	8	10
Ni(NCS)_2_ · 2(INH-DCB)	10	12	9	11
Ni(ClO_4_)_2_ · 3(INH-DCB)	9	11	8	10
Streptomycin	16	18	16	18

**Table 8:** Fungicidal screening data of INH-DCB and its Co(II) and Ni(II) complexes.

Compound	Percentage inhibition after 96 h (conc in ppm)					
	*F oxysporum*			*M phaseolina*		
	50	100	200	50	100	200

INH-DCB	41	50	55	40	50	55
CoCl_2_ · 2(INH-DCB)	44	51	57	42	55	59
CoBr_2_ · 2(INH-DCB)	44	50	57	43	54	61
Co(NO_3_)_2_ · 2(INH-DCB)	43	51	56	44	56	62
Co(NCS)_2_ · 2(INH-DCB)	48	56	61	47	56	63
Co(CH_3_COO)_2_ · 2(INH-DCB)	45	54	60	45	55	60
Co(ClO_4_)_2_ · 3(INH-DCB)	44	50	57	45	56	60
NiCl_2_ · 2(INH-DCB)	43	49	54	43	51	57
NiBr_2_ · 2(INH-DCB)	44	49	54	43	52	57
Ni(NO_3_)_2_ · 2(INH-DCB)	44	49	55	44	53	56
Ni(NCS)_2_ · 2(INH-DCB)	47	56	62	48	58	63
Ni(ClO_4_)_2_ · 3(INH-DCB)	45	54	59	44	53	57
Bavistin	84	100	100	80	99	100

## References

[1] Ainscough EW, Brodie AM, Ranford JD, Waters JM (1995). Hexafluorosilicate coordination to the antitumour
copper(II) salicylaldehyde benzoylhydrazone
(H_2_L) system: single-crystal X-ray structure of
[{Cu (HL) H_2_O}_2_ SiF_6_] .2H_2_O. *Inorganica Chimica Acta*.

[2] Koh LL, Kon OL, Loh KW (1998). Complexes of salicylaldehyde acylhydrazones: cytotoxicity,
QSAR and crystal structure of the sterically hindered t-butyl dimer. *Journal of Inorganic Biochemistry*.

[3] Ainscough EW, Brodie AM, Denny WA, Finlay GJ, Gothe SA, Ranford JD (1999). Cytotoxicity of salicylaldehyde benzoylhydrazone analogs
and their transition metal complexes: quantitative structure-activity relationships. *Journal of Inorganic Biochemistry*.

[4] Küçükgüzel G, Rollas S, Küçükgüzel I, Kiraz M (1999). Synthesis and antimycobacterial activity of some coupling
products from 4-aminobenzoic acid hydrazones. *European Journal of Medicinal Chemistry*.

[5] Yang Z-Y, Yang R-D, Li F-S, Yu K-B (2000). Crystal structure and antitumor activity of some rare
earth metal complexes with Schiff base. *Polyhedron*.

[6] Bottari B, Maccari R, Monforte F, Ottanà R, Rotondo E, Vigorita MG (2000). Isoniazid-related copper(II) and nickel(II) complexes with
antimycobacterial in vitro activity. Part 9. *Bioorganic & Medicinal Chemistry Letters*.

[7] Sridhar SK, Saravanan M, Ramesh A (2001). Synthesis and antibacterial screening of hydrazones,
Schiff and Mannich bases of isatin derivatives. *European Journal of Medicinal Chemistry*.

[8] Koçyiğit KB, Rollas S (2002). Synthesis, characterization and evaluation of
antituberculosis activity of some hydrazones. *Il Farmaco*.

[9] Kakimoto S, Yamamoto K (1956). Studies on antitubercular compounds. X.
Condensation products of aldehydes and acid hydrazides of pyridine group. *Pharmaceutical Bulletin*.

[10] Aggarwal RC, Singh NK, Singh RP (1979). Synthesis and structural studies of first row transition
metal complexes of salicylaldehyde hydrazone. *Inorganica Chimica Acta*.

[11] Anten JA, Nicholls D, Markopoulos JM, Markopoulou O (1987). Transition-metal complexes of hydrazones derived from
1,4-diformyl- and 1,4-diacetylbenzenes. *Polyhedron*.

[12] Tossidis IA, Bolos CA, Aslanidis PN, Katsoulos GA (1987). Monohalogenobenzoylhydrazones III. Synthesis and structural
studies of Pt(II), Pd(II) and Rh(III) complexes of
Di-(2-pyridyl)ketonechlorobenzoyl hydrazones. *Inorganica Chimica Acta*.

[13] Maiti A, Ghosh S (1989). Synthesis and reactivity of some octa coordinated
dioxouranium(VI) complexes of diacetyl bis(benzoyl-hydrazone) and benzil
bis(benzoyl hydrazone). *Indian Journal of Chemistry*.

[14] Agarwal RK, Garg P, Agarwal H, Agarwal SK (1997). Synthesis, magneto-spectral and thermal studies of
cobalt(II) and nickel(II) complexes of
4[N-(4-dimethylaminobenzalidene) amino]antipyrine. *Synthesis and Reactivity in Inorganic and Metal-Organic Chemistry*.

[15] Welcher FJ (1965). *The Analytical Uses of Ethylenediamine Tetraacetic Acid*.

[16] Vogel AI (1978). *A Textbook of Quantitative Inorganic Analysis*.

[17] Kurz E, Kober G, Berl M (1958). Determination of perchlorates by fusion with nitrite. *Analytical Chemistry*.

[18] Zehr EI, Bird GW, Fisher KD (1978). *Methods for Evaluating Plant Fungicides,
Nematicides and Bactericides*.

[19] Geary WJ (1971). The use of conductivity measurements in organic solvents
for the characterisation of coordination compounds. *Coordination Chemistry Reviews*.

[20] Schlapp R, Penney WG (1932). Influence of crystalline fields on the susceptibilities
of salts of paramagnetic ions. II. The iron group, especially Ni, Cr and Co. *Physical Review*.

[21] Figgis BN, Nyholm RS (1958). A convenient solid for calibration of the Gouy
susceptibilitity apparatus. *Journal of Chemical Society*.

[22] Kato M, Jonassen HB, Fanning JC (1964). Copper(II) complexes with subnormal magnetic moments. *Chemical Reviews*.

[23] Yamada S (1966). Recent aspects of the stereochemistry of Schiff-base-metal complexes. *Coordination Chemistry Reviews*.

[24] Usha (1996). *Synthesis and characterization of transition
metal complexes of semicarbazones and thiosemicarbazones *[PhD thesis].

[25] Nakamoto K (1970). *Infrared Spectra of Inorganic and Coordination Compounds*.

[26] Agarwal RK, Sarin RK (1993). Synthesis and characterization of some lanthanide(III)
perchlorato complexes of hydrazones of isonicotinic acid hydrazide. *Polyhedron*.

[27] Agarwal RK, Sarin RK, Agarwal H (1995). Magneto, spectral studies on some lanthanide(III)
nitrates and isothiocyanates complexes of hydrazones of isonicotinic acid hydrazide. *Bulletin of the Chemical Society of Ethiopia*.

[28] Bellamy LJ (1954). *The Infrared Spectra of Complex Molecules*.

[29] Burns GR (1968). Metal complexes of thiocarbohydrazide. *Inorganic Chemistry*.

[30] Swaminathan K, Irving HMNH (1964). Infra-red absorption spectra of complexes of thiourea. *Journal of Inorganic and Nuclear Chemistry*.

[31] Hathaway BJ, Underhill AE (1961). The infrared spectra of some transition-metal perchlorates. *Journal of the Chemical Society*.

[32] Radhakrishnan PS, Indrasenan P (1989). Synthesis and characterization of some lanthanide
perchlorate and nitrate complexes of 4-(pyridine-3-carboxalidene)aminoantipyrine. *Indian Journal of Chemistry*.

[33] Agarwal RK, Prakash J (1991). Synthesis and characterization of thorium(IV) and
dioxouranium(VI) complexes of 4-[N(2-hydroxy-1-naphthalidene)amino]antipyrine. *Polyhedron*.

[34] Agarwal RK, Dutt P, Prakash J (1992). Synthesis and characterization of thorium(IV)
and dioxouranium(VI) complexes of 4-[N-(m-methoxybenzylidene)amino]antipyrine. *Polish Journal of Chemistry*.

[35] Kumar Y, Sethi PD, Jain CL (1990). Spectral, magnetic, mossbauer and chemotherapeutical
studies of iron(III) complexes with various new derivatives
of isonicotinic acid hydrazide. *Journal of Indian Chemical Society*.

[36] Ross SD (1962). Forbidden transitions in the infra-red spectra of
some tetrahedral anions—I. Perchlorates. *Spectrochimica Acta*.

[37] Ramamurthy P, Patel CC (1964). Pyridine N-oxide complexes of zirconyl,
thorium and uranyl perchlorates. *Canadian Journal of Chemistry*.

[38] Mitchell PCH, Williams RJP (1960). The infrared spectra and general properties of inorganic thiocyanates. *Journal of the Chemical Society*.

[39] Bailey RA, Michelsen TW, Mills WN (1971). Observations on the i.r. intensity criterion for
the bonding mode in thiocyanate complexes. *Journal of Inorganic and Nuclear Chemistry*.

[40] Clark RJH (1965). Metal-halogen stretching frequencies in inorganic complexes. *Spectrochimica Acta*.

[41] Burmeister JL (1966). Recent developments in the coordination chemistry of
ambidentate ligands. *Coordination Chemistry Reviews*.

[42] Burmeister JL (1968). Linkage isomerism in metal complexes. *Coordination Chemistry Reviews*.

[43] Burmeister JL (1990). Ambidentate ligands, the schizophrenics of
coordination chemistry. *Coordination Chemistry Reviews*.

[44] Karayannis NM, Mikulski CM, Pytlewski LL, Labes MM (1974). 2-, 3-, 4- picoline N-oxide complexes with cobalt(II),
nickel(II) and copper(II) nitrates. *Inorganic Chemistry*.

[45] Addison CC, Logan N, Wallwork SC, Garner CD (1971). Structural aspects of co-ordinated nitrate groups. *Quarterly Reviews of the Chemical Society*.

[46] Heter RE, Grossman WEL (1966). Vibrational analysis of bidentate nitrate and carbonate complexes. *Inorganic Chemistry*.

[47] Ferraro JR (1960). The nitrate symmetry in metallic nitrates. *Journal of Molecular Spectroscopy*.

[48] Devi GS, Indrasenan P (1987). Uranyl nitrate complexes of some Schiff bases of
4-aminoantipyrine and certain carbonyl compounds. *Inorganica Chimica Acta*.

[49] Agarwal RK, Prasad S (2005). Synthesis, spectroscopic and physicochemical
characterization and biological activity of
Co(II) and Ni(II) coordination compounds of
4-aminoantipyrine thiosemicarbazone. *Bioinorganic Chemistry and Applications*.

[50] Casellato U, Vigato PA, Vidali N (1978). Actinide complexes with carboxylic acids. *Coordination Chemistry Reviews*.

[51] Ahuja IS, Yadav CL, Tripathi S (1989). Coordination polymers of some uranyl salts involving
4,4′-bipyridyl, 4,4′-bipyridyl N,N′-dioxide, 1,3-bis(4-pyridyl)
propane and hexamethylenetetramine. *Asian Journal of Chemistry*.

[52] Tanabe Y, Sugano S (1954). On the absorption spectra of complex ions. I. *Journal of the Physical Society of Japan*.

[53] Tanabe Y, Sugano S (1954). On the absorption spectra of complex ions. II. *Journal of the Physical Society of Japan*.

[54] Lever ABP (1968). Electron spectra of some transition metal complexes.
Derivation of Dq and B. *Journal of Chemical Education*.

[55] Reedijk J, Driessen WL, Groeneveld WL (1969). A semi-empirical energy-level diagram for octahedral cobalt(II) complexes. *Recueil des Travaux Chimiques des Pays-Bas*.

